# Supplementary material for: Assessment of knowledge and preventive behavior regarding rubella virus among females in Riyadh, Saudi Arabia: A cross-sectional study
Source: PLoS One. 2025 May 7;20(5):e0306156. doi: 10.1371/journal.pone.0306156 (PMC12058167; doi:10.1371/journal.pone.0306156)
Supplement: Attachment — Submitted filename: report.docx [file pone.0306156.s002.docx]

**Reviewer report for PLOS one Journal**

Title: Assessment of Knowledge, Attitude, and Preventive Behavior Regarding Rubella Virus

Among Females in Riyadh, Saudi Arabia

1. **Abstract:**
   1.1. In abstract, the method section is poor, it is not well written. It lacks important data such as statistical analysis, study variables, and population.
   1.2. The result section in abstract is deficient and very poor, it is not supported by enough findings. It was written as if it is conclusion, no values or interpretations found.
   1.3. The authors mentioned in result section that (Awareness of rubella's symptoms, like fever and rash, is common) and (Vaccination awareness and uptake are low).These findings are not supported by number or percentage.
   1.4. In abstract, conclusion section is poor, it does not represent the findings.
2. **Introduction:** suitable and expressive.

The objectives are clear, and so the presentation

3. **Methods :** the methods are well described. The deficient point that the authors did not mention how the attitude is assessed. Under the section **2.4.** *Study Questionnaire Development*, the authors described various parts of the survey without writing anything about the attitude although it was a main part in the title of the manuscript.
4. **Results section:**

This part is poor. The analysis is not satisfied. The authors did not write the assessment of attitude among the participants. I have observed the following deficient points:

4.1. The authors did not obtain the score levels of knowledge and attitude
4.2. The authors did not make comparisons among the participants such as males and females, marital status, education levels, and so on
4.3. The authors did mention the factors affecting the Knowledge, Attitude, and Preventive Behavior Regarding Rubella Virus.

**I suggest the followings:**

4.4. The authors should obtain score levels for Knowledge, Attitude, and Preventive Behavior, and compare the mean/median levels among the participants using chi-square test
4.5. The authors should add a regression table to demonstrate the factors that influence the Knowledge, Attitude, and Preventive Behavior Regarding Rubella Virus

5. **Interpretation:**  Unsatisfied
6. **Conclusion:** Unsatisfied
