# Supplementary material for: Assessment of knowledge and preventive behavior regarding rubella virus among females in Riyadh, Saudi Arabia: A cross-sectional study
Source: PLoS One. 2025 May 7;20(5):e0306156. doi: 10.1371/journal.pone.0306156 (PMC12058167; doi:10.1371/journal.pone.0306156)
Supplement: Attachment — Submitted filename: Response to Reviewers.docx [file pone.0306156.s003.docx]

**Reviewer report for PLOS one Journal**

Title: Assessment of Knowledge, Attitude, and Preventive Behavior Regarding Rubella Virus

Among Females in Riyadh, Saudi Arabia

Dear Reviewer 1,

Thank you for your thoughtful feedback and for recognizing the significance of our research on the Rubella virus, specifically concerning the Assessment of Knowledge, Attitude, and Preventive Behavior Regarding the Rubella Virus Among Females in Riyadh, Saudi Arabia. We appreciate your interest in our study and your constructive comments.

We acknowledge your suggestions regarding the manuscript's reporting and agree that improvements can be made to enhance clarity and comprehensiveness. We are committed to refining the manuscript to better present our findings and ensure that the information is accessible and valuable to the readers.

We carefully reviewed your specific points and incorporated the necessary revisions to address the areas of concern. This will include a more detailed explanation of our methodology, a clearer presentation of our results, and a more in-depth discussion of the implications of our findings.

Thank you once again for your valuable input. We are resubmitting a revised version that meets the high standards of the journal and contributes meaningfully to the field of public health research.

1. **Abstract:**
   1.1. In abstract, the method section is poor, it is not well written. It lacks important data such as statistical analysis, study variables, and population.

Thank you for your feedback. I have revised the abstract to include key data on statistical analysis, study variables, and population details. These additions aim to provide a clearer overview of the study's scope and findings.

1.2. The result section in abstract is deficient and very poor, it is not supported by enough findings. It was written as if it is conclusion, no values or interpretations found.

Thank you for your feedback. I've updated the abstract's results section to include more specific findings, including key values and interpretations, to better support the conclusions.

1.3. The authors mentioned in result section that (Awareness of rubella's symptoms, like fever and rash, is common) and (Vaccination awareness and uptake are low). These findings are not supported by number or percentage.

Thank you for your feedback. I've revised the results section to include findings supported by percentages for greater clarity and specificity.

1.4. In the abstract, the conclusion section is poor, it does not represent the findings.

Thank you for your feedback. I've updated the conclusion section in the abstract to clearly represent the findings of the study.

2. **Introduction:** suitable and expressive.

The objectives are clear, and so the presentation

3. **Methods :** the methods are well described. The deficient point that the authors did not mention how the attitude is assessed. Under the section **2.4.** *Study Questionnaire Development*, the authors described various parts of the survey without writing anything about the attitude although it was a main part in the title of the manuscript.

Thank you for your feedback. I've now included an assessment of attitudes and provided a detailed description of the survey's focus on attitudes, which aligns with the main theme of the manuscript.

4. **Results section:**

This part is poor. The analysis is not satisfied. The authors did not write the assessment of attitude among the participants. I have observed the following deficient points:

4.1. The authors did not obtain the score levels of knowledge and attitude

Thank you for your feedback. I've added the score levels for knowledge and attitude in the manuscript for better clarity and context.

4.2. The authors did not make comparisons among the participants such as males and females, marital status, education levels, and so on

I would like to inform the reviewer that this manuscript does not include male participants, which means that we are unable to make comparisons between the female and male participants.

The reason: Understanding and improving women's awareness and preventive behaviors is crucial because rubella infection during pregnancy can cause Congenital Rubella Syndrome (CRS). To optimise public health efforts to prevent Rubella transmission and its complications in new-borns, health education and intervention strategies should target females, the population most at risk of adverse outcomes. While men also prevent disease, the direct impact on pregnancy outcomes justifies the focus on women in this assessment.

4.3. The authors did mention the factors affecting the Knowledge, Attitude, and Preventive Behavior Regarding Rubella Virus.

Thank you for your feedback. I have now included a section detailing the factors affecting Knowledge, Attitude, and Preventive Behavior regarding the Rubella Virus. The factors affecting the knowledge, attitude, and preventive behavior regarding the Rubella virus among females in Riyadh include significant gaps in understanding Rubella, with many participants being aware of the virus but lacking detailed knowledge about its transmission and complications, particularly during pregnancy. Misconceptions about transmission routes, such as airborne droplets and contaminated surfaces, contribute to this knowledge gap. Attitudes are influenced by varying levels of concern about the spread of Rubella and uncertainty about its severity and personal susceptibility. Preventive behaviors are hindered by limited vaccination awareness and uptake, driven by concerns about vaccine safety, lack of awareness, and healthcare access issues. However, consistent hygiene practices and peer support for vaccination indicate a positive trend in preventive behavior among the community.

**I suggest the following:**

4.4. The authors should obtain score levels for Knowledge, Attitude, and Preventive Behavior, and compare the mean/median levels among the participants using chi-square test.

Thank you for your feedback. I would like to clarify that comparing mean/median levels among participants using the chi-square test is not feasible because the survey was designed to emphasize qualitative aspects rather than quantitative measures. This approach limits the applicability of statistical tests intended for quantitative data.

4.5. The authors should add a regression table to demonstrate the factors that influence Knowledge, Attitude, and Preventive Behavior Regarding Rubella Virus.

Thank you for your feedback. I have included a paragraph detailing the factors influencing Knowledge, Attitude, and Preventive Behavior regarding the Rubella Virus.

5. **Interpretation:**  Unsatisfied.

Thank you for your valuable feedback. The updated interpretation now provides a more comprehensive and satisfying. I appreciate your insights and believe these changes enhance the clarity and depth of the discussion.

6. **Conclusion:** Unsatisfied.

Thank you for your thoughtful comments. I have revised the Conclusion section to address your concerns and provide a more satisfying summary of the findings. I believe these changes better capture the key points and implications of the study.
