# Supplementary material for: Assessment of knowledge and preventive behavior regarding rubella virus among females in Riyadh, Saudi Arabia: A cross-sectional study
Source: PLoS One. 2025 May 7;20(5):e0306156. doi: 10.1371/journal.pone.0306156 (PMC12058167; doi:10.1371/journal.pone.0306156)
Supplement: Attachment — Submitted filename: Response_to_Reviewers_auresp_2.docx [file pone.0306156.s005.docx]

**Reviewer report for PLOS one Journal**

Title: Assessment of Knowledge, Attitude, and Preventive Behavior Regarding Rubella Virus

Among Females in Riyadh, Saudi Arabia

Dear Editor,

I would like to sincerely thank you for your efforts in evaluating my manuscript. However, I have a concern regarding the use of artificial intelligence (AI) in the reviewing process. I understand that your journal does not allow researchers to use AI in their manuscripts, yet I was surprised to find that some reviewers may have used AI to assess my work. Some comments seem to indicate this, such as:

A reviewer questioned the phrase: "in the Department of Pathology, College of Medicine, Imam Mohammad Ibn Saud Islamic University, Riyadh, Saudi Arabia" — asking "Why the department of pathology?" This phrasing is specific to my affiliation and seemed to be misunderstood.

Another reviewer commented: "The authors did not make comparisons among the participants such as males and females." However, my manuscript does not include male participants, which makes such comparisons impossible.

While I have taken on board much of the constructive feedback and made all appropriate revisions, I am concerned about the apparent use of AI in reviewing, especially given the journal's stance on this issue.

I hope these concerns will be considered and that my manuscript will be accepted for publication, as I believe it offers valuable insights for specialists in this field.

Thank you for your time and consideration.

Best regards,

Dr. Jehad Aldali

Dear Reviewer 1,

Thank you for your thoughtful feedback and for recognizing the significance of our research on the Rubella virus, specifically concerning the Assessment of Knowledge, Attitude, and Preventive Behavior Regarding the Rubella Virus Among Females in Riyadh, Saudi Arabia. We appreciate your interest in our study and your constructive comments.

We acknowledge your suggestions regarding the manuscript's reporting and agree that improvements can be made to enhance clarity and comprehensiveness. We are committed to refining the manuscript to better present our findings and ensure that the information is accessible and valuable to the readers.

We carefully reviewed your specific points and incorporated the necessary revisions to address the areas of concern. This will include a more detailed explanation of our methodology, a clearer presentation of our results, and a more in-depth discussion of the implications of our findings.

Thank you once again for your valuable input. We are resubmitting a revised version that meets the high standards of the journal and contributes meaningfully to the field of public health research.

1. **Abstract:**
   1.1. In abstract, the method section is poor, it is not well written. It lacks important data such as statistical analysis, study variables, and population.

Thank you for your feedback. I have revised the abstract to include key data on statistical analysis, study variables, and population details. These additions aim to provide a clearer overview of the study's scope and findings.

1.2. The result section in abstract is deficient and very poor, it is not supported by enough findings. It was written as if it is conclusion, no values or interpretations found.

Thank you for your feedback. I've updated the abstract's results section to include more specific findings, including key values and interpretations, to better support the conclusions.

1.3. The authors mentioned in result section that (Awareness of rubella's symptoms, like fever and rash, is common) and (Vaccination awareness and uptake are low). These findings are not supported by number or percentage.

Thank you for your feedback. I've revised the results section to include findings supported by percentages for greater clarity and specificity.

1.4. In the abstract, the conclusion section is poor, it does not represent the findings.

Thank you for your feedback. I've updated the conclusion section in the abstract to clearly represent the findings of the study.

2. **Introduction:** suitable and expressive.

The objectives are clear, and so the presentation

3. **Methods :** the methods are well described. The deficient point that the authors did not mention how the attitude is assessed. Under the section **2.4.** *Study Questionnaire Development*, the authors described various parts of the survey without writing anything about the attitude although it was a main part in the title of the manuscript.

Thank you for your feedback and for pointing out the questionnaire's attitude information gaps. We appreciate your thoughtful suggestion.

After thinking about it, the current data does not cover the attitude dimension enough. The manuscript has been revised accordingly. In particular, we removed "attitude" from the title and objectives to focus on knowledge and prevention.

The revised title is now "Assessment of Rubella Virus Knowledge and Preventive Behavior Among Females in Riyadh, Saudi Arabia".

We adjusted the objectives to match this new focus.

Thank you again for your constructive feedback, which improved our manuscript's clarity and focus.Thank you for your feedback and for pointing out the questionnaire's attitude information gaps. We appreciate your thoughtful suggestion.

4. **Results section:**

This part is poor. The analysis is not satisfied. The authors did not write the assessment of attitude among the participants. I have observed the following deficient points:

4.1. The authors did not obtain the score levels of knowledge and attitude

Thank you for your suggestion. We have removed "attitude" from the title and objectives, as recommended. Following this revision, we no longer need to obtain the score levels for knowledge and attitude. The manuscript now focuses solely on the assessment of knowledge and preventive behavior regarding Rubella.

4.2. The authors did not make comparisons among the participants such as males and females, marital status, education levels, and so on

I would like to inform the reviewer that this manuscript does not include male participants, which means that we are unable to make comparisons between the female and male participants.

The reason: Understanding and improving women's awareness and preventive behaviors is crucial because rubella infection during pregnancy can cause Congenital Rubella Syndrome (CRS). To optimise public health efforts to prevent Rubella transmission and its complications in new-borns, health education and intervention strategies should target females, the population most at risk of adverse outcomes. While men also prevent disease, the direct impact on pregnancy outcomes justifies the focus on women in this assessment.

4.3. The authors did mention the factors affecting the Knowledge, Attitude, and Preventive Behavior Regarding Rubella Virus.

Thank you for your feedback. I have now included a section detailing the factors affecting Knowledge and Preventive Behavior regarding the Rubella Virus. The factors affecting the knowledge and preventive behavior regarding the Rubella virus among females in Riyadh include significant gaps in understanding Rubella, with many participants being aware of the virus but lacking detailed knowledge about its transmission and complications, particularly during pregnancy. Misconceptions about transmission routes, such as airborne droplets and contaminated surfaces, contribute to this knowledge gap. Preventive behaviors are hindered by limited vaccination awareness and uptake, driven by concerns about vaccine safety, lack of awareness, and healthcare access issues. However, consistent hygiene practices and peer support for vaccination indicate a positive trend in preventive behavior among the community.

**I suggest the following:**

4.4. The authors should obtain score levels for Knowledge, Attitude, and Preventive Behavior, and compare the mean/median levels among the participants using chi-square test.

Thank you for your feedback. I would like to clarify that comparing mean/median levels among participants using the chi-square test is not feasible because the survey was designed to emphasize qualitative aspects rather than quantitative measures. This approach limits the applicability of statistical tests intended for quantitative data.

4.5. The authors should add a regression table to demonstrate the factors that influence Knowledge, Attitude, and Preventive Behavior Regarding Rubella Virus.

Thank you for your feedback. I have included a paragraph detailing the factors influencing Knowledge, Attitude, and Preventive Behavior regarding the Rubella Virus.

5. **Interpretation:**  Unsatisfied.

Thank you for your valuable feedback. The updated interpretation now provides a more comprehensive and satisfying. I appreciate your insights and believe these changes enhance the clarity and depth of the discussion.

6. **Conclusion:** Unsatisfied.

Thank you for your thoughtful comments. I have revised the Conclusion section to address your concerns and provide a more satisfying summary of the findings. I believe these changes better capture the key points and implications of the study.

Reviewer #3: 1) Many grammar and editing errors 
 rubella --- r should be capital in the abstract and whole manuscript, but many are unclear about how it spreads.

Thank you for your valuable feedback. I have carefully reviewed your suggestion, and I have corrected the usage of "rubella" throughout the manuscript and abstract by capitalizing the "R" to ensure consistency and proper scientific nomenclature.

2) The objectives should be smart (person, time, place) in the abstract and the whole manuscript (add the time).

3) in the abstract---------- The study included a total of 448 participants, all of whom were females (replace to be) (of 448 females (aged -------).

I appreciate your feedback. As requested in your comments, I revised the sentences. If adjustments are needed, please let me know.

4) In the abstract, An online survey using Google Forms in English and translated into Arabic was used to record information----- an online Arabic survey using Google Forms --- no need to this detail in the abstract .

I appreciate your feedback. As requested in your comments, I revised the sentences. If adjustments are needed, please let me know.

5) The term gender is a behavioral term, not a physiological term, so it replaces sex in the whole manuscript.

I appreciate your feedback. As requested in your comments, I revised the sentences. If adjustments are needed, please let me know.

6)  The combination of fever and skin rash was the most frequently reported symptom, accounting for 23.4% of all cases. ----- cases of rubella ----- Add a number before the %.

I appreciate your input. The document has been revised to include the number prior to the %, as per your recommendation.

7)  Regarding the serious health risk, 55.4% of the participants were unsure of the situation.---- What does it mean I don't know or 
Thank you for your valuable feedback. I have carefully reviewed your suggestion, and I have corrected the sentences more clear, the severity of the health threat, 55.4% of participants indicated they were uncertain or unsure about the seriousness of the situation. This suggests a significant portion of the group may lack clarity or confidence in their understanding of the health risks involved, highlighting a potential area for further education or communication efforts.
8)  in certain areas---- females, women, cases, participants fix the term in the whole manuscript ---- to be females as in the title

Thank you for your feedback. I have addressed your suggestion and revised the term throughout the entire manuscript. Please let me know if any further adjustments are needed.
------ in the Kingdom of Saudi Arabia and  Saudi Arabia-----fix the term in the whole manuscript to be as in the title.

Thank you for your feedback. I have addressed your suggestion and revised the term throughout the entire manuscript. Please let me know if any further adjustments are needed.

-----This study aims to assess the level of awareness, attitudes,------- knowledge in others ---fix the term in the whole manuscript to be as in the title.

Thank you for your feedback. I have addressed your suggestion and revised the term throughout the entire manuscript. Please let me know if any further adjustments are needed.

9)  The KAP survey , US  -- add the full name for the first time.

Thank you for your valuable feedback. I have added the full name for the term upon its first mention in the manuscript, as you suggested. Please let me know if you have any further recommendations.

10) in the abstract --- . Questions were included in the questionnaire to evaluate awareness of rubella, knowledge of symptoms, transmission, and preventative measures of rubella infection--------- where is the attitude 
and the awarness is part of the knowledge

Thank you for your feedback and for pointing out the questionnaire's attitude information gaps. We appreciate your thoughtful suggestion.

After thinking about it, the current data does not cover the attitude dimension enough. The manuscript has been revised accordingly. In particular, we removed "attitude" from the title and objectives to focus on knowledge and prevention.

The revised title is now "Assessment of Rubella Virus Knowledge and Preventive Behavior Among Females in Riyadh, Saudi Arabia".

11) you discuss the global incidence in numbers in details ----- and The impact of this program over the years has been significant. The incidence of Rubella in Saudi Arabia has dramatically declined since the initiation of 
the vaccination program (17).------------------------ kindly add the numbers in detail.

Thank you for your feedback. I would like to inform you that the global incidence in numbers in details in the discussion section.

12) add a brief about the   initiation of the vaccination program --- in Saudi Arabia (started when, achievement)---in the introduction

Thank you for your feedback. I would like to inform you that the initiation of the vaccination program, particularly for Rubella in Saudi Arabia, it was written in the introduction section.


13)  in the Department of Pathology, collage of Medicine, Imam Mohammad Ibn Saud Islamic University, Riyadh, Saudi Arabia.------- why the department of pathology ???

**I would like to inform you that the following sentences are exclusive to my affiliation.**

14) In the Riyadh province of Saudi Arabia regional the sample size of 385 participants was 
sufficient to achieve 95% confidence with a 5% margin of error.----- as a  cross sectional study you need a prevlance or incidence (with refrence) --- and the total popoulation or females in Riyadh.

The total popoulation or females in Saudi Arabia around nine million, in light of this. sample size of 385 participants was sufficient to achieve 95% confidence with a 5% margin of error.

15)The second section focused on the information the Knowledge about rubella, specifically interested in their comprehension of the disease, their perception of the modes of transmission of rubella, their level of concern regarding it, their perspectives on the gravity of the virus, and their awareness of the impact of this virus on pregnant women and their embryos. The third section focused on information about the symptoms caused by rubella. immunization status.  The last section focuses on preventive measures against rubella. -----  add refrence ???.

I appreciate your input. I would like to inform you that the methods section in the abstract is a summary of the methods, so there is no need for references.

16) The third section focused on information about the symptoms caused by rubella—its knowledge, Rubella immunization status----- It is a part of preventive measures (rewrite).

I appreciate your input. I would like to inform you that rewrite the third section include Rubella immunization status with preventive measures

18) while women comprise 96.2%.--- what does it mean

19) the age groups should be continous , and in order in table 1
25-<30
30-<40
40-<50
under 18-<25
years > 50

Your feedback is greatly appreciated. I would like to emphasize that the age groups listed in Table 1 are sequential and logical.

why the age intervals is not fixed ( 7 y then 4-year -10 year).

Thank you for your comment. Now the age groups presented in Table 1 are continuous and organized in ascending order. If you have any further questions or need additional information, please feel free to ask.

20) in all tables should be not Effective.

Thank you for your comment. The tables now is effective.

21) in knowledge study ---- should be total knowledge score
where does it then classify groups s that a fair number of participants are known of the rubella virus, but many are unclear about how it spreads---- what is fair mean

24) Table 5: Strategies for preventing rubella ---- should be the practice of preventive measure including vaccination status

25) where is the attitude tables ?

Thank you for your feedback and for pointing out the questionnaire's attitude information gaps. We appreciate your thoughtful suggestion.

After thinking about it, the current data does not cover the attitude dimension enough. The manuscript has been revised accordingly. In particular, we removed "attitude" from the title and objectives to focus on knowledge and prevention.

The revised title is now "Assessment of Rubella Virus Knowledge and Preventive Behavior Among Females in Riyadh, Saudi Arabia".
