# Supplementary material for: Assessment of knowledge and preventive behavior regarding rubella virus among females in Riyadh, Saudi Arabia: A cross-sectional study
Source: PLoS One. 2025 May 7;20(5):e0306156. doi: 10.1371/journal.pone.0306156 (PMC12058167; doi:10.1371/journal.pone.0306156)
Supplement: Attachment — Submitted filename: Response To the Reviewers.docx [file pone.0306156.s006.docx]

Dear Editor

I hope this message finds you well. I would like to express my sincere gratitude for your care and guidance regarding my manuscript, [Manuscript Title].

I am pleased to inform you that I have carefully addressed all the reviewers' comments and suggestions. The revised version has been submitted for your consideration. I truly appreciate the valuable feedback provided by the reviewers, which has greatly enhanced the quality of the manuscript.

Thank you once again for your time and effort in overseeing this process. I look forward to hearing your thoughts on the revised submission.

Best Regards,

Dear Reviewer 4,

I hope this message finds you well. I am writing to express my heartfelt gratitude for your time, effort, and valuable feedback in reviewing my manuscript. I truly appreciate your constructive comments and suggestions, which have undoubtedly contributed to enhancing the quality of my work.

I am delighted to know that opinion for my manuscript to accepted for publication, and I am sincerely thankful for your support in this process. Your expertise and insights have been invaluable.

Please do not hesitate to reach out if there is any additional information or further revisions required. Once again, thank you for your kind support and for playing such a crucial role in this achievement.

Warm regards,

Dear Reviewer 5

I hope this message finds you well. Following your valuable feedback, I have carefully revised the abstract of my manuscript to make it clearer and more concise.

The updated version reflects your suggestions and aims to enhance the overall clarity and readability of the content. I deeply appreciate your insights and guidance, which have significantly improved the quality of the manuscript.

Please let me know if there are any additional adjustments you would recommend or further steps I should take. Thank you once again for your time and expertise.

Best regards,

Dear Reviewer 6

I hope this message finds you well. I am writing to inform you that I have completed a thorough proofreading of my manuscript to ensure it is more polished and professional. This step was taken to eliminate any grammatical errors and enhance the overall readability and presentation.

I believe the revised version aligns with the high standards expected for publication, and I sincerely thank you for your valuable feedback, which motivated me to refine the manuscript further.

Please feel free to share any additional suggestions or areas for improvement. I greatly appreciate your time and dedication in reviewing my work.

Best regards,
