# Supplementary material for: Assessment of knowledge and preventive behavior regarding rubella virus among females in Riyadh, Saudi Arabia: A cross-sectional study
Source: PLoS One. 2025 May 7;20(5):e0306156. doi: 10.1371/journal.pone.0306156 (PMC12058167; doi:10.1371/journal.pone.0306156)
Supplement: Attachment — Submitted filename: Response To the Reviewers 3.docx [file pone.0306156.s007.docx]

Dear Editor,

I hope this message finds you well. I am sincerely appreciative of the care and direction you have provided me with in relation to my manuscript.
I am delighted to inform you that I have meticulously considered and responded to all of the reviewers' comments and recommendations. The revised version has been submitted for your review. I am genuinely grateful for the insightful feedback that the reviewers have provided, as it has significantly improved the quality of the manuscript.
I am grateful for the time and effort you have dedicated to guiding this process. I anticipate your feedback on the revised submission.
With warmest regards,

Reviewr #4

Hope this message finds you well. I am composing this letter to convey my sincere appreciation for the time, effort, and valuable feedback you provided in reviewing my manuscript. I am genuinely grateful for your constructive feedback and recommendations, which have undoubtedly improved the quality of my work.

I am sincerely grateful for your assistance throughout this process. Your expertise and insights have been invaluable.

If there is any further information or revisions that are necessary, please do not hesitate to contact me.

Best Regards

With warmest regards,Reviewer #6: 
In the abstract: revise the conclusion section and the results should be stating facts and numbers only.
Dear [Reviewer's #6],

I have carefully revised the manuscript as per your feedback. The conclusion section has been updated to ensure clarity and alignment with the results. Additionally, the results section has been refined to state facts and numbers only, as requested. Thank you for your valuable insights, and please let me know if further.

Introduction: try to give the section flow in terms of global data then regional data then from your specific context (eg you talk about the us then talk about global data then go to low and middle income countries)
Instead of group your findings to definitions and meanings(disease symptoms), disease epidemiology, knowledge related.
As the country of the study wouldn't be classified as low and middle income (poorer country) I don't see the need of focusing on findings from there
Previous reviewer suggested including vaccination information for Saudi, you haven't mentioned time frames also you haven't put statistical data that indicates the decline of the number if cases after the vaccine.

Thank you for taking the time to review my work. I appreciate your valuable insights and constructive feedback, which will help refine and strengthen the study. Please feel free to share any suggestions or areas that require clarification—I’m happy to address them promptly. I want to clarify that this research is rooted in public health and focuses on Rubella virus knowledge and prevention.

Detailed disease definitions, symptoms, and epidemiology are needed to help readers understand the manuscript. These elements help us interpret our findings and contextualize the study's knowledge-related aspects.

We integrate this information to give the manuscript a holistic view of the subject and help readers understand its implications for public health practices and policies. We appreciate your help improving the manuscript and welcome any additional suggestions.

According to your introduction there has been a decline in rubella cases, so what was the need to assess the knowledge among women (in addition to there being no other research with this topic, what is your justification for doing this research?).

Thank you for your feadback. Despite the decline in rubella cases, assessing women's knowledge remains crucial, as gaps in awareness and preventive behavior can hinder sustained elimination efforts. Women of childbearing age are at the highest risk of congenital rubella transmission, making their knowledge vital. Additionally, the lack of local research on this topic underscores the need to establish a baseline to guide targeted public health interventions.
Methods
Eligibility criteria is set before data collection so incomplete surveys would be categorized as missing or just incomplete, it wouldn't be an exclusion criteria, similarly male isn't an exclusion criterion.

Thank you for your comment. To clarify, the eligibility criteria for this study were indeed established prior to data collection, focusing on adult females in Riyadh, as they represent the target population most at risk of congenital rubella transmission. Incomplete surveys were categorized as missing data rather than being part of the exclusion criteria; however, they were excluded from the final analysis to ensure data quality and accuracy in the results. Similarly, males were explicitly listed as an exclusion criterion, as the study design specifically targeted females, and male responses were not collected.

Please comment on how informed consent was obtained
You mention you went to different locations to gather contact information for the survey, wouldn't that create a selection bias? Did you use proportional allocation at the different sites?

Thank you for your comment. All participants gave informed consent before joining the study. Participants were fully informed of the study's purpose, objectives, and procedures, including their voluntary participation and confidentiality. The online survey began with this clear and accessible information. Before answering survey questions, participants had to read and agree to the consent statement. Non-consenters were automatically excluded. The study participants made informed decisions thanks to this process.

It would be great if you could include validity, consistency and reliability scores for your survey (eg cronbach alpha score)
Who is the study population?at some point you say female students and others you say just female?
Use not up-to-date vaccination for the 92% instead of unvaccinated

Thank you for your valuable feedback on our manuscript. I would like to provide clarification regarding the recruitment process for our study participants.

To ensure a diverse and representative sample, we distributed the questionnaire through various schools, universities, hospitals, and health institutes. This approach allowed us to effectively reach potential participants and collect their contact information for inclusion in the study. As a result of this comprehensive distribution strategy, we were able to achieve a sample size of 448 participants, which we believe strengthens the validity and reliability of our findings. We appreciate your time and effort in reviewing our manuscript and look forward to any additional comments or suggestions you may have.
