# Supplementary material for: Assessment of knowledge and preventive behavior regarding rubella virus among females in Riyadh, Saudi Arabia: A cross-sectional study
Source: PLoS One. 2025 May 7;20(5):e0306156. doi: 10.1371/journal.pone.0306156 (PMC12058167; doi:10.1371/journal.pone.0306156)
Supplement: Attachment — Submitted filename: Plos One Review Comments.docx [file pone.0306156.s008.docx]

**Title and Abstract: Abstract is clear and concise**

**Introduction: Introduction is enough if any current study mentioned that would be better.**

**Methodology**

1- **Study Settings and Design**

- **Please explain why do you choose the cross-sectional over Case control or longitudinal studies.**
- **Could you explain the data collection locations in Riyadh city did you cover major part of the city.**

2- **Study Subjects**

- **What was the study subjects recruitment methods?**
- **Clearly write the inclusion and exclusion criteria of the subjects.**

3- **Sample Size**

- **Clearly write why the final sample size (447) of the subjects are more than the calculated size (385).**

4- **Questionnaire Development**

- **Please write how did you validated the Questionnaire, what was statistical measures were used to see the reliability of the Questionnaire?**
- **Pilot results were seen on a very small sample size, like only 5 faculties were selected for this, which is very small size, there should be minimum 7 or more subjects for this pilot testing.**

5- **Ethical Issues**

- **Ethical approval was taken, but how did you obtained the informed consent?**

6- **Results Section**
1- **Demographic Features**

- **What was the reason for younger and more qualified subjects were more aware about rubella? You need to do more analysis to see these demographics effects on knowledge.**
- **Can you discuss more about the single participants that is 62.3% are notable, does the marriage status have any effect on attitude towards rubella?**

2- **Knowledge about Rubella**

- **Study findings shows that only 2.1% thinks that they are Very knowledgeable, discuss what is the reason for that?**

3- **Vaccination History**

- **Very low vaccination rate that is 7.5% received rubella vaccine that is a point of concern, it should be discussed in discussion, weather it is due to the healthcare accessibility, cultural or lack of awareness programs.**
- **Another findings that 47% subjects were unaware weather they have taken rubella vaccine, this is also point of concern, why it is so, do the primary healthcare centers do not communicate to the general public.**

4- **Discussion section**

**Knowledge Spaces**

- **What do you feel, why there significant gaps in knowledge about the rubella complications and transmission, discuss potential reasons for that.**
- **You can discuss more on the similar studies done in Saudi Arabia in different regions and compare it with your findings.**

**Vaccination Awareness**

- **Discuss and see other studies in Saudi Arabia and you can suggest how to come up with this low vaccinated rate in Riyadh.**

**Cultural and Social Reasons**

- **You can discuss whether some cultural belief, or misconceptions preventing subjects going for rubella vaccine.**

**Suggestions for Public Health**

- **You can suggest specific recommendations for healthcare providers to start educational campaigns about the transmission and complications of rubella.**

**References**

- **Most of the references are old and before 2020, it would be better to new studies done in Saudi Arabia or Middle East.**

**Overall the study has covered many aspects of rubella, after taking above points, manuscript will be off great contribution for rubella awareness, knowledge, prevention and control the complication and its transmission in public.**
