# Supplementary material for: Assessment of knowledge and preventive behavior regarding rubella virus among females in Riyadh, Saudi Arabia: A cross-sectional study
Source: PLoS One. 2025 May 7;20(5):e0306156. doi: 10.1371/journal.pone.0306156 (PMC12058167; doi:10.1371/journal.pone.0306156)
Supplement: Attachment — Submitted filename: Plos_One_Review_Comments_auresp_5.docx [file pone.0306156.s009.docx]

Dear Editor,

After careful consideration, I feel that the manuscript has merit and it does fully meet PLOS ONE’s publication criteria as it currently stands. The study is well-conceived and executed, and I believe it will make a valuable contribution to the field. I am confident in recommending its publication. We would like to note that all comments from the reviewer in Research 11 have been addressed. The only addition made to the plan was the suggestion provided by the reviewer.

Sincerely,

Dear Reviewer 4,

I hope this message finds you well. I am writing to express my heartfelt gratitude for your time, effort, and valuable feedback in reviewing my manuscript. I truly appreciate your constructive comments and suggestions, which have undoubtedly contributed to enhancing the quality of my work.

I am delighted to know that opinion for my manuscript to accepted for publication, and I am sincerely thankful for your support in this process. Your expertise and insights have been invaluable.

Please do not hesitate to reach out if there is any additional information or further revisions required. Once again, thank you for your kind support and for playing such a crucial role in this achievement.

Warm regards,

**Reviewer 11**

Hope this message finds you well. After your helpful feedback, I carefully revised my manuscript abstract to make it clearer and more concise.

Your comments I answer most of them and I improved the content's clarity and readability in the updated version. I appreciate your advice, which greatly improved the manuscript.

Please let me know if there are any additional adjustments you would recommend or further steps I should take. Thank you once again for your time and expertise.

**Methodology**

1- **Study Settings and Design**

- **Please explain why do you choose the cross-sectional over Case control or longitudinal studies.**

**Response to the reviewer:**

Thank you for your insightful comments on our study, Our goal was to assess rubella virus knowledge and prevention among Riyadh women at a specific time, so we chose a cross-sectional study design. This design is more practical than a longitudinal study because it quickly captures awareness and practices without long-term follow-up. Our focus was on public understanding and prevention, not disease incidence, unlike a case-control study, which is ideal for comparing exposures and outcomes in diseased and non-diseased groups. We efficiently collected data using the cross-sectional approach, which informed public health interventions.

- **Could you explain the data collection locations in Riyadh city did you cover major part of the city.**

**Response to the reviewer:**

Thank you for your feedback. We would like to clarify that our manuscript already addresses the data collection locations in the **Participants** section. Specifically, we stated: "The study targeted females from various educational institutions in Riyadh. The research team visited various schools, universities, hospitals, and health institutes to collect the contact information of potential participants." This approach ensured that our sample included individuals from diverse backgrounds and different parts of Riyadh, enhancing the generalizability of our findings

**Study Subjects**

- **What was the study subjects recruitment methods?**

**Response to the reviewer:**

I appreciate your kind comments. Our study recruited female participants from Riyadh hospitals, educational institutions, and health institutes. The research team introduced the study, distributed information, and collected contact information at these locations. Participants were then invited to take the survey in person or online. This method allowed us to efficiently reach a diverse group of females in educational and healthcare settings, ensuring broad representation while maintaining feasibility within the study timeline.

- **Clearly write the inclusion and exclusion criteria of the subjects.**

**Response to the reviewer:**

I appreciate your feedback. Please note that the manuscript clearly stated study participant inclusion and exclusion criteria. We set eligibility criteria to include only relevant participants in the study.

2- **Sample Size**

- **Clearly write why the final sample size (447) of the subjects are more than the calculated size (385).**

**Response to the reviewer:**

Thank you for your thoughtful comment. To account for incomplete responses and ensure a more robust data set, the final sample size (447 participants) exceeded the calculated desired sample size (385 participants). This method reduced missing or unusable data and improved the validity and representativeness of the study. The slightly larger sample size also improved the statistical power of the subgroup analysis**.**

3- **Questionnaire Development**

- **Please write how did you validated the Questionnaire, what was statistical measures were used to see the reliability of the Questionnaire?**

**Response to the reviewer:**

Thank you for your thoughtful comment. To ensure the reliability of the questionnaire beyond expert reviews and pilot studies, we utilized a previously validated survey from a published scientific study, making modifications where necessary to fit our study context. Initially, the revised questionnaire was reviewed by five specialists in human medicine to assess its relevance, clarity, and accuracy. Following their feedback, it was further evaluated by five non-specialist doctors to ensure comprehensibility for a broader audience. Their comments were carefully considered, and necessary refinements were made to enhance clarity, minimize ambiguity, and improve the consistency of responses, thereby strengthening the overall reliability of the survey.

- **Pilot results were seen on a very small sample size, like only 5 faculties were selected for this, which is very small size, there should be minimum 7 or more subjects for this pilot testing.**

**Response to the reviewer:**

Your thoughtful comment is appreciated. We modified a validated survey from a published scientific study to fit our study context to ensure the questionnaire's reliability beyond expert reviews and pilot studies. Five human medicine experts reviewed the revised questionnaire for relevance, clarity, and accuracy. After their feedback, five non-specialist doctors reviewed it for general comprehension. Their suggestions were carefully considered and refined to improve clarity, minimize ambiguity, and improve response consistency, thereby strengthening the survey's reliability.

4- **Ethical Issues**

- **Ethical approval was taken, but how did you obtained the informed consent?**

**Response to the reviewer:**

I appreciate your comment. We followed ethical approval guidelines by submitting a complete research proposal with study objectives, methodology, and ethical considerations. We also submitted the survey questionnaire and a detailed informed consent form to disclose the study's purpose, voluntary participation, confidentiality, and data protection. This process protected participants' rights and ethical standards.

5- **Results Section**
1- **Demographic Features**

- **What was the reason for younger and more qualified subjects were more aware about rubella? You need to do more analysis to see these demographics effects on knowledge.**

**Response to the reviewer:**

I appreciate your comment. According to literature, higher education and recent health information increase rubella awareness, so younger and more qualified participants were more aware. The current results support this conclusion, so we believe demographic effects on knowledge do not require further analysis.

- **Can you discuss more about the single participants that is 62.3% are notable, does the marriage status have any effect on attitude towards rubella?**

**Response to the reviewer:**

Thank you for your valuable feedback. We acknowledge that the current data does not sufficiently cover the attitude dimension. Accordingly, we have revised the manuscript to reflect this, ensuring that attitude is not included in the title or objectives. Our focus remains on knowledge and preventive behavior regarding rubella. We appreciate your insight and believe these revisions enhance the clarity and alignment of our study.

2- **Knowledge about Rubella**

- **Study findings shows that only 2.1% thinks that they are Very knowledgeable, discuss what is the reason for that?**

**Response to the reviewer:**

I appreciate your comment. We clarify that Table 2 in the manuscript shows participants' rubella knowledge. As shown, 57.6% of participants knew about rubella, while 42.4% did not. These findings show a significant awareness gap, emphasizing the need for rubella public health education.

3- **Vaccination History**

- **Very low vaccination rate that is 7.5% received rubella vaccine that is a point of concern, it should be discussed in discussion, weather it is due to the healthcare accessibility, cultural or lack of awareness programs.**

**Response to the reviewer:**

Thank you for your comment. We would like to clarify that in the Discussion section of the manuscript, we have addressed the assessment of vaccinations as an integral part of understanding the preventive behaviors and potential barriers among females in Riyadh. Specifically, we mentioned that lack of disease knowledge, cultural or religious misconceptions, limited healthcare access, vaccine side effects, and misinformation could act as barriers to vaccination. We emphasized that addressing these barriers can help inform targeted public health interventions to improve awareness and vaccination uptake.

- **Another findings that 47% subjects were unaware weather they have taken rubella vaccine, this is also point of concern, why it is so, do the primary healthcare centers do not communicate to the general public.**

**Response to the reviewer:**

Thank you for your thoughtful comment. In our manuscript, we acknowledged that 48.4% of participants were unaware of whether they had received the rubella vaccine, highlighting a significant public health concern. We discussed that lack of awareness, concerns about vaccine safety, and limited access to healthcare are key reasons for this uncertainty. While primary healthcare centers play a crucial role in public health communication, our findings suggest that gaps in vaccine record-keeping, insufficient public health outreach, and misinformation may contribute to this lack of awareness. Strengthening vaccination awareness campaigns and improving communication between healthcare providers and the public could help address this issue. We hope this clarification answers your concern.

4- **Discussion section**

**Knowledge Spaces**

- **What do you feel, why there significant gaps in knowledge about the rubella complications and transmission, discuss potential reasons for that.**

**Response to the reviewer:**

Thank you for your insightful comment. In our manuscript, we highlighted the significant gaps in knowledge regarding rubella’s complications and transmission among participants. Specifically, many were unaware that rubella can spread through airborne droplets or contaminated surfaces, with most attributing transmission solely to direct contact. Furthermore, there was considerable uncertainty about the severity of rubella-related complications, particularly the risks it poses to pregnant women and their fetuses.

These gaps may stem from several factors, including insufficient public health education campaigns, limited emphasis on rubella in routine health communication, and a lack of accessible, reliable information about the disease’s transmission and complications. Additionally, low perceived susceptibility to rubella could contribute to the participants’ limited knowledge. Addressing these issues through targeted public health interventions and improved community outreach is essential to bridge these knowledge gaps and promote preventive behaviors.

- **You can discuss more on the similar studies done in Saudi Arabia in different regions and compare it with your findings.**

**Response to the reviewer:**

Thank you for your comment. In our manuscript, we have discussed similar studies conducted in different regions of Saudi Arabia and compared their findings with ours. However, we acknowledge that research on rubella awareness, transmission, and preventive behaviors in Saudi Arabia remains limited, particularly in certain regions. This scarcity of data highlights the need for further studies to better understand the public’s knowledge and attitudes toward rubella nationwide. Our findings contribute to filling this gap and provide valuable insights that can inform future public health strategies.

**Vaccination Awareness**

- **Discuss and see other studies in Saudi Arabia and you can suggest how to come up with this low vaccinated rate in Riyadh.**

**Response to the reviewer:**

I appreciate your feedback. We compared our findings to those of other Saudi Arabian studies in our manuscript. There is little research on rubella vaccination rates in the country, but our findings show a low rate in Riyadh, which may be due to lack of awareness, vaccine hesitancy, misinformation, and limited healthcare access.

We recommend improving public health education campaigns about rubella and its complications, integrating rubella vaccine awareness into routine maternal and child health programs, and improving primary healthcare center vaccination record-keeping and accessibility. Targeted community engagement and healthcare provider communication to address vaccine safety myths may also increase vaccination rates. These strategies and strengthened national immunization policies may help Riyadh increase rubella vaccine uptake.

**Cultural and Social Reasons**

- **You can discuss whether some cultural belief, or misconceptions preventing subjects going for rubella vaccine.**

**Response to the reviewer:**

Thanks for your thoughtful comment. In our manuscript, we acknowledge that cultural beliefs and misconceptions may prevent some from receiving the rubella vaccine. Misinformation about vaccine safety, side effects, and infertility or other health risks can cause vaccine hesitancy. Lack of emphasis on adult vaccination, especially for non-pregnant women, may lower uptake.

Community-based awareness campaigns, trusted healthcare providers and religious leaders, and clear public health messaging to dispel myths and promote rubella vaccination are needed to overcome these barriers. We appreciate your suggestion and recognize the importance of addressing cultural and societal factors to boost vaccination rates.

**Suggestions for Public Health**

- **You can suggest specific recommendations for healthcare providers to start educational campaigns about the transmission and complications of rubella.**

**Response to the reviewer:**

Thank you for your valuable suggestion. In response to your feedback, we have added specific recommendations in the manuscript for healthcare providers to initiate educational campaigns on rubella transmission and complications. These recommendations include community-based health seminars, integration of rubella education into routine healthcare visits, social media awareness campaigns, collaboration with religious and community leaders, school and university programs, and specialized training for healthcare professionals. These initiatives aim to enhance public awareness and promote higher vaccination uptake.

**References**

- **Most of the references are old and before 2020, it would be better to new studies done in Saudi Arabia or Middle East.**

**Response to the reviewer:**

I appreciate your feedback. Many of our manuscript's references are from before 2020. This is due to the lack of recent Saudi Arabian and Middle Eastern rubella awareness, transmission, and vaccination studies. Due to the lack of updated regional research, we included relevant older studies that provide foundational insights. We also used recent global literature to support our discussion.
